# mRNA and Peptide Vaccines in Melanoma—Current Landscape and Future Direction

**DOI:** 10.3390/cells15040344

**Published:** 2026-02-13

**Authors:** Jiaxing Jason Qin, Yang Wang, Shahneen Sandhu

**Affiliations:** 1 Department of Medical Oncology, Peter MacCallum Cancer Centre, Melbourne, VIC 3000, Australia; jason.qin@petermac.org (J.J.Q.); zhe.wang@petermac.org (Y.W.); 2 Sir Peter MacCallum Department of Oncology, University of Melbourne, Melbourne, VIC 3010, Australia

**Keywords:** mRNA vaccines, melanoma, tumour immunology, peptide vaccines, neoadjuvant therapy

## Abstract

Immune checkpoint inhibitors have transformed the treatment landscape for advanced melanoma in the past 15 years, delivering unprecedented and durable survival benefits. This success has propelled the development of complementary immune-directed therapies, including cancer vaccines. Among these, synthetic long peptide (SLP) and mRNA vaccine platforms have emerged as highly promising. Advances in next-generation sequencing technology, alongside computational neoantigen algorithm predictions, have enabled patient-specific neoantigen identification to improve vaccine immunogenicity and enhance therapeutic efficacy. Off-the-shelf and personalised SLP and mRNA vaccines have demonstrated the ability to induce robust antigen-specific T-cell responses and modulate the tumour microenvironment. Mechanistically, cancer vaccines synergise with immune checkpoint inhibition. This review outlines the current clinical development of mRNA and peptide vaccines in melanoma, highlighting the significant promise to synergise with immune checkpoint inhibition to enhance efficacy without adding to the systemic toxicity profile. The neoadjuvant setting, characterised by intact tumour antigens and draining lymphatic architecture, offers a compelling biological context for leveraging cancer vaccines for enhanced immune priming and response assessment. Collectively, the rapid advances in technology and emerging clinical data position cancer vaccines as a promising therapy capable of improving immunotherapy in Stage III and IV melanoma.

## 1. Introduction

Melanoma is the most aggressive form of skin cancer and is a leading cause of death from cutaneous malignancies [1]. The advent of immune checkpoint inhibitors (ICIs) has dramatically transformed outcomes, with up to approximately half of all patients diagnosed with advanced melanoma now achieving long-term survival beyond 10 years [2]. In Stage III melanoma, the use of neoadjuvant (before surgery) and adjuvant (after surgery) ICIs has demonstrated marked benefits for relapse-free survival (RFS) [3,4,5,6]. The remarkable success of ICIs is underpinned by the inherent immunogenicity of melanoma, driven by high tumour mutation burden (TMB), abundant tumour infiltrating lymphocytes (TILs), and high expression of tumour-associated antigens (TAAs). These characteristics continue to propel the development of the next generation of immune-directed therapies, including cancer vaccines, adoptive cellular therapies such as engineered melanoma-specific T-cell receptors (TCRs) and TILs, and therapies targeting specific cytokine receptors such as CCR2, CCR4, IL-6, and IL-15, among others.

Vaccination has long been a cornerstone of infectious disease prevention and typically involves the inoculation of a molecular preparation to elicit durable protective immunity [7]. A similar concept underlies cancer vaccines, whereby tumour-derived antigens are used as inoculants to stimulate an adaptive T-cell response capable of recognising and eliminating tumour cells. Firstly, tumour antigens are taken up and processed by professional antigen-presenting cells (APCs), primarily dendritic cells, and presented on major histocompatibility complex (MHC) II molecules, which activate naïve CD4+ T-cells by binding with TCRs and co-stimulatory receptor activation between CD28 on T-cells and CD80/86 on APCs. This activation induces cytokines, including IL-2, IFN-ꝩ and lymphotoxin, which prime and sustain antigen-specific cytotoxic T-cells and macrophages, as well as promote antibody production via B-cells and memory T-cell formation. Antigen cross-presentation on MHC I molecules by APCs activates effector CD8+ T-cells to recognise tumour cells displaying tumour antigens and to exert cytotoxic effects through perforin and granzyme-mediated apoptosis [8,9,10]. While non-professional APCs can also present antigens via MHC I and engage CD8+ T-cells, the absence of concomitant CD4+ T-cell activation via MHC class II limits broader adaptive immunity, and consequently, the overall immune response tends to be less sustained and is prone to tolerance [9,11,12].

The identification of relevant tumour antigens presented by APCs is central to cancer vaccine development. TAAs are self-antigens that are aberrantly expressed or overexpressed in tumour cells but present on normal cells at lower levels. In contrast, tumour-specific antigens (TSAs, also known as neoantigens) are self-antigens that arise from somatic genetic mutations during tumorigenesis and are exclusively expressed by tumour cells [13,14].

Despite decade-long efforts, early cancer vaccines yielded limited clinical efficacy due to weak immunogenicity and failure to overcome immune tolerance [7,15,16]. However, recent advances, including the advent of ICIs, synthetic long peptide (SLP) vaccine platforms and messenger RNA (mRNA) vaccines, have rekindled significant interest. Early clinical data have shown that these vaccines, particularly when combined with ICIs, can elicit potent antitumour responses as adjuncts in combination therapies.

Historically, peptide vaccines encoding short segments (8–11 amino acids) of tumour antigens elicited limited tumour-specific immune responses. Their efficacy was constrained by MHC I restriction, incomplete T-cell activation, and susceptibility to rapid enzymatic degradation, leading to poor cytotoxic T-cell responses and the emergence of immune tolerance. In contrast, SLPs, typically 11–30 amino acids in length, encoding multiple epitopes, are presented on both MHC I and MHC II molecules by professional and non-professional APCs, enabling the activation of both CD4+ and CD8+ immune responses that promote a more robust and durable antitumour response [11,12,14,15,17].

mRNA vaccine technology, accelerated by the COVID-19 pandemic, has further revolutionised the therapeutic vaccine landscape. Contemporary mRNA vaccine technology enables rapid, scalable and cost-effective production of mRNA sequences that encode antigens which can be delivered safely to patients to induce strong antigen-specific immune responses [18,19,20]. Both SLP and mRNA vaccine platforms allow for the inclusion of multiple TAAs or TSAs within a single vaccine formulation to overcome human leukocyte antigen (HLA) restriction and antigen heterogeneity across sites while enhancing antigen presentation through both MHC I and II pathways [12].

Beyond direct T-cell activation, emerging evidence suggests that vaccines may also reshape the tumour microenvironment (TME) to improve immune recognition of tumour cells and response to immune-directed therapies. The immunosuppressive TME is mediated by the recruitment of suppressive cells, including FoxP3+ regulatory T-cells (Treg), which suppress effector T-cell responses by secretion of immunosuppressive molecules, including TGF-β, IL-8 and IL-10 or direct cell contact [21,22]. Tumour cell surface expression of immunosuppressive ligands, such as programmed death-ligand 1 (PD-L1), also actively inhibits cytotoxic T-cell responses. The expression of cytotoxic T-lymphocyte-associated protein 4 (CTLA-4) on the T-cell surface is another inhibitory mechanism by which the T-cell response is suppressed [22]. Blockade of PD-1/PD-L1 or CTLA-4 using ICIs has demonstrated remarkable antitumour activities in melanoma, and the combination of anti-PD-1 and anti-CTLA-4 ICIs showed improved efficacy compared to single-agent ICI [2]. However, a certain proportion of melanoma patients exhibit primary resistance to ICIs, and a proportion of patients who initially responded to ICIs subsequently develop adaptive resistance. The mechanisms behind primary and adaptive resistance are thought to be associated with the high Treg-to-effector T-cell ratio and the expression of immunosuppressive cytokines in the TME, downregulation of tumour antigen presentation, effector T-cell exhaustion, and development of escape mutation variants in tumour cells [22]. Preclinical investigations suggest vaccines, especially mRNA vaccines, were able to increase the release of proinflammatory cytokines, including TNF-α and IFN-ꝩ, induce CD4+ and CD8+ T-cell infiltration, and promote tumour antigen expression, thereby reshaping the TME from being immunosuppressive to immune sensitive and potentially overcoming primary and adaptive resistance to immune-directed therapies. In addition, combination vaccine and ICI therapies appear to induce a stronger anti-tumoural response than either therapy alone in preclinical models, suggesting a synergistic effect and supporting further investigation of combination therapy approaches [23,24,25,26]. Collectively, SLP and mRNA vaccines represent a new generation of immunotherapies that can potentially propel cancer vaccines into mainstream clinical application.

This review aims to provide an overview of the mechanistic and technological foundations underpinning mRNA and SLP vaccine development in melanoma, as well as the immunological and clinical evidence supporting adjuvant and neoadjuvant immune-directed therapies. It also explores opportunities, benefits and challenges of incorporating mRNA and peptide vaccines into standard treatment paradigms for melanoma.

## 2. Tumour Antigens in Cancer Vaccines

Many cancer cells express peptides that are released in the bloodstream or presented on the cell surface by MHC molecules. Peptides recognised by the immune system that are capable of eliciting an immune response are considered antigenic, including TAAs and TSAs. TSAs are unique self-antigens that arise as a consequence of either viral oncoproteins or somatic genomic mutations generating neoantigens. These include single-nucleotide variants (SNVs), single-nucleotide base insertions or deletions (INDELs), gene fusions, aberrant RNA splicing, disordered post-translational modification, and integrated viral open reading frames (ORF) [27]. Viral oncoprotein-associated TSAs, such as those found in human papillomavirus-associated cervical cancer and Epstein–Barr virus-associated nasopharyngeal cancer, are created by virally encoded ORFs [28]. Among somatic mutations, SNVs account for most of the nonsynonymous neoantigen peptides. These peptides, generated during proteasomal degradation of aberrant proteins and presented by MHC molecules on the surface of tumour cells, can elicit an antigen-specific immune response [29].

A minority of neoantigens, arising from mutations in oncogenes like PIK3CA, RAS and BRAF, may be shared across patients in multiple cancer types [30]. INDEL mutations generate nonsynonymous ORFs, producing neoantigens that are often highly immunogenic and display greater binding affinity to MHC I [31]. INDEL frequency also reflects the mutagenic aetiology of the tumour; for example, higher numbers of INDELS are observed in cutaneous melanoma compared with mucosal melanoma, reflecting ultraviolet (UV)-induced DNA damage [32]. Gene fusions represent another mechanism for neoantigen generation through chromosomal translocations or inversions and can elicit a potent cytotoxic T-cell response. In patients with melanoma treated with anti-PD-1, responders showed a reduction in the number of fusion neoantigens (2.22 vs. 0.67; *p* = 0.019), whereas no numerical difference was observed in tumours that were stable (2.29 vs. 1.71; *p* = 0.32) or progressing (2.53 vs. 2.43; *p* = 0.94) on treatment [30,33]. This data suggests that immune surveillance may selectively eliminate tumours carrying fusion-derived neoantigens.

Cutaneous melanoma exhibits one of the highest rates of TMB, often exceeding 100 mutations per megabase, largely due to UV exposure [34]. UV radiation leads to impairment in DNA damage repair mechanisms, promoting genomic instability and mutagenesis. Melanoma cells express high levels of TAAs, such as the melanoma antigen gene (MAGE) family antigens and cancer testis antigens such as NY-ESO-1 [35]. Additionally, the high TMB confers a high number of tumour-specific neoantigens that are readily recognised by the immune system. Multiple studies have demonstrated high neoantigen load in melanoma (9 to 16,300 neoantigens per tumour), and this has been shown to correlate with ICI responsiveness [36,37]. Snyder et al. [37] demonstrated that shared tetrapeptide neoepitopes were enriched amongst patients deriving durable benefit from CTLA-4 blockade, supporting the persistence of antigen-driven memory after expanded TILs.

For cancer vaccines to be efficacious, the selection of target antigens is crucial. The ideal antigens should be abundantly expressed on tumour cells, exhibit no or minimal expression in normal tissues, and be sufficiently distinct from host antigens to overcome immunological tolerance and mitigate the risk of autoimmunity. Historically, melanoma vaccines have focused on highly expressed TAAs and embryonic antigens such as ganglioside GM2, glycoprotein-100 (gp-100), preferentially expressed antigen in melanoma (PRAME), and MAGE family antigens, as well as restricted gangliosides including GM2, GD2, GT3 and 9-O-Ac-GD3 [38]. Despite strong preclinical data, vaccines directed against TAAs have historically yielded limited clinical efficacy, likely due to the rapid emergence of immune tolerance and tumour antigen heterogeneity [15,30,39]. Furthermore, the application of this technology has been constrained by the limited accuracy of antigen prediction due to the vast heterogeneity of TMB and distant antigen presentation among tumour types [40].

Recent advances in bioinformatics, next-generation sequencing (NGS), RNA sequencing and proteomics have accelerated the identification of immunogenic neoantigens, paving the way for personalised cancer vaccines [15]. Importantly, research is now expanding beyond MHC class I-restricted neoantigens that induce CD8+ T-cell responses to include MHC class II-binding neoantigens that induce CD4+ T-cell-mediated regulation of antitumour immunity. Moreover, nonspecific mRNA vaccines designed to boost T-cell activation in conjunction with ICI therapy are also showing promise. Recent preclinical data indicate mRNA vaccines can upregulate IFN-ꝩ and augment cytokine-mediated T-cell recruitment and increase PD-L1 expression on tumours, rendering immunologically “cold” tumours more sensitive to concurrent ICIs, leading to improved survival outcomes [24,41].

## 3. Cancer Vaccine Delivery Platforms: mRNA and Peptides

Beyond antigen selection, the vaccine delivery platform also represents an important consideration. An ideal vaccine should be straightforward and cost-effective to manufacture, possess stability with an extended shelf life, be applicable across diverse clinical settings, and elicit strong immune activation with minimal toxicity. Broadly, vaccine delivery strategies can be categorised as cellular-based, peptide and mRNA technologies. Cellular-based vaccines involve the ex vivo loading of APCs, most commonly dendritic cells, with tumour antigens, followed by reintroduction of these antigen-loaded APCs to the host to stimulate a tumour-specific T-cell response. This process involves the harvesting of APCs via leukapheresis, ex vivo maturation, activation, antigen loading and subset selection, all of which are technically demanding, costly and limited by a short shelf life, restricting use to selected specialist centres and clinical settings [17,42,43].

mRNA transfers genetic information from DNA to ribosomes, where it is synthesised into proteins. The applicability of this technology is expanding, as it can be used for antigen expression, gene editing, and protein therapies against infectious diseases or for cancer treatment [20,44]. The development of mRNA vaccines for tumour prevention dates back to the 1970s, when Langer et al. demonstrated that nucleic acids could be encapsulated and delivered by biocompatible polymers [45]. Advances in chemical modification and purification of mRNA, as well as improvements in drug delivery, such as lipid, polymeric and hybrid nanoparticles, have improved the in vivo viability and delivery of mRNA [46,47,48,49]. The COVID-19 pandemic accelerated this field, demonstrating production scalability and improving immunogenicity, resulting in the success of mRNA vaccines such as the Moderna mRNA-1273 and the Pfizer/BioNTech BNT162b2, with others in early-phase clinical development (Moderna mRNA-4359) [50,51,52]. mRNA vaccines offer several advantages, such as encoding multiple antigens to be presented by APCs across diverse HLA types, reducing the potential for HLA restriction, and broadening T-cell activation [18,53,54]. Unlike DNA-based therapies, mRNAs do not integrate into the host genome, eliminating the risk of insertional mutagenesis [19]. The transient expression and rapid degradation by normal cellular processes reduce the risk of potential autoimmunity [17].

**Figure 1:**
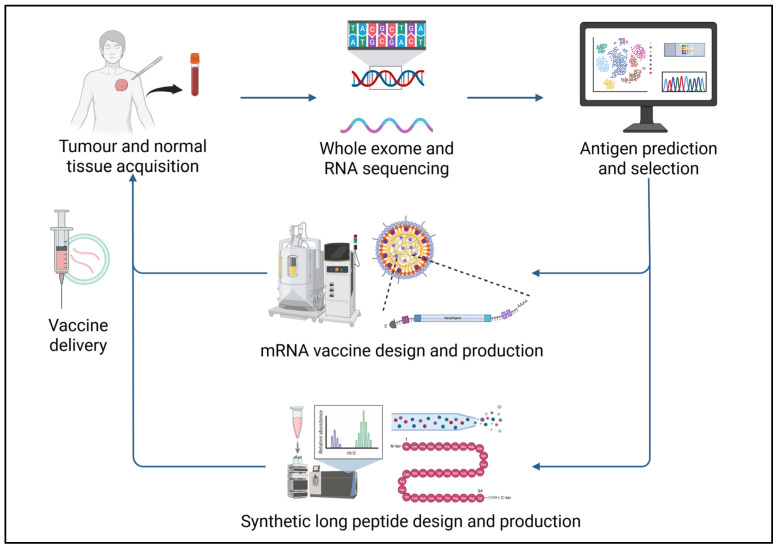
Diagrammatic representation of the mRNA and SLP vaccine manufacturing process. Figure created using Biorender^®^.

The manufacture of mRNA vaccines begins with the identification and selection of target antigens (Figure 1). In the case of a neoantigen vaccine, nonsynonymous mutations that are tumour-specific are first identified through comparative whole exome and RNA sequencing of tumour tissues and normal tissues and prioritised based on predicted MHC class I and II binding affinity [53]. The selected RNA sequences are incorporated into a synthetic mRNA construct comprising a 5′ cap to add stability, and together with 5′ and 3′ untranslated regions, an open reading frame, and a poly(A) tail to enhance stability and translation efficiency [18,55].

Key challenges with mRNA vaccines include the inherent instability of mRNA, large molecular size, negative charge and susceptibility to enzymatic degradation [47,56]. To improve mRNA vaccine stability and delivery, the mRNA molecules are encapsulated in delivery vehicles, most commonly lipid nanoparticles (LNPs), to prevent enzymatic degradation and improve organ- or tissue-specific delivery [55,57]. LNPs also exhibit additional immunostimulant activity, triggering the release of cytokines such as IL-6, IL-12 and IL-1β and upregulating co-stimulatory toll-like receptor agonists (TLRs), including TLR4, to augment the immunogenicity of the vaccine [58]. Once administered, the mRNA molecules are delivered to the cytoplasm of APCs, where tumour antigens are translated, processed and presented on MHC class I and II molecules.

Peptide vaccine synthesis has also benefitted from significant technological advances, making long-chain peptide synthesis and purification techniques relatively straightforward and cost-effective [59]. SLPs are assembled from amino acids by stepwise deprotection and coupling on a solid support such as resin, with downstream purification using high-performance liquid chromatography and mass spectrometry [59]. These advances enable scalable production of multi-epitope SLP vaccine constructs that can activate diverse T-cell clones to induce a more robust immune response and mitigate immune tolerance. Once administered, the long peptides are internalised, processed and presented by APCs on MHC I and II molecules to elicit both CD4+ and CD8+ immune responses [12]. Peptide vaccines are flexible and precise in antigen design and have high tumour specificity, low risk of autoimmunity, and a good safety profile [60].

To further enhance immunogenicity, adjuvants are incorporated into mRNA or peptide vaccines to activate innate and adaptive immunity. Adjuvants are generally classified into three categories: (1) RNAs with intrinsic adjuvant properties, (2) components of the delivery system, and (3) exogenous immunostimulants [61]. Unmodified exogenous mRNA, such as that contained within mRNA vaccines, has intrinsic adjuvant activity and can activate pattern recognition receptors, including TLR3, TLR7 and TLR8, leading to cytokine release, including TNF-α, IL-6, IL-12, IL-1β and IFNα/β [61]. Exogenous immunostimulants, including arginine-rich protamine peptides, may be added to vaccines to further activate TLR7/8 pathways, promoting B- and T-cell responses and suppressing circulating FoxP3+/CD4+ Treg cells, as demonstrated in an early-phase trial [62,63]. Ongoing research is focused on optimising adjuvant formulations that further improve immunogenicity without causing excessive reactogenicity [64].

## 4. Clinical Development of mRNA and SLP Vaccines

Clinical application of mRNA and SLP vaccines in melanoma is increasingly garnering interest, with multiple trials evaluating efficacy, safety and optimal therapeutic settings (Table 1).

### 4.1. mRNA Vaccines

In a first-in-human Phase I study, a personalised mRNA vaccine encoding ten potentially immunogenic neoantigens was investigated in patients with Stage III and IV melanoma. All 13 patients inoculated intra-nodally with the vaccine demonstrated a strong poly-neo-epitope T-cell response against the encoded neoantigens and tolerated the treatment well [65]. In another Phase I study, a personalised mRNA vaccine encoding up to 20 neoantigens encapsulated in a lipoplex formulation (autogene cevumeran) was investigated as either a monotherapy or in combination with atezolizumab, an anti-PD-L1 antibody, in patients with locally advanced or metastatic solid tumours, including melanoma. Despite poor baseline clinical characteristics, including exposure to multiple lines of prior therapy, high burden of metastatic disease, and low PD-1 expression, approximately 70% of the patients mounted de novo amplification of T-cell responses against at least one of the neoantigens encoded by their mRNA vaccine, irrespective of whether the vaccine was administered as a monotherapy or in combination with atezolizumab. All melanoma patients received the combination therapy. Among the T-cell responses seen, 59% induced CD4+ responses only, 26% induced CD8+ responses only and 15% induced both CD4+ and CD8+ responses. In ICI-experienced melanoma patients (*n* = 8), 75% maintained stable disease with a median progression-free survival (mPFS) of 4.0 months and a median overall survival (mOS) of 20 months. In ICI-naïve melanoma patients (*n* = 9), the overall response rate (ORR) was 33.3%, with an mPFS of 1.4 months and mOS not reached [66].

In the Phase IIb KEYNOTE-942 study, patients with fully resected Stage IIIB–IV melanoma were randomised to receive a personalised mRNA vaccine encoding up to 34 neoantigens in an LNP formulation delivered intramuscularly in combination with pembrolizumab, an anti-PD-1 antibody, vs. pembrolizumab alone. The ICI vaccine combination demonstrated a higher RFS (HR 0.561; 95% CI 0.309–1.017; *p* = 0.053) and distant metastasis-free survival (MFS) (HR 0.347; 95% CI 0.145–0.828; *p* = 0.013) regardless of TMB compared with pembrolizumab monotherapy. Distant MFS rate at 18 months was 91.8% in the combination arm versus 76.8% in the pembrolizumab monotherapy arm (HR 0.347; 95% CI 0.145–0.828; *p* = 0.0063). The safety profile was favourable, with 83% (86/104) experiencing Grade 1–2 treatment-related adverse events (TRAEs). Common vaccine-related AEs were fatigue in 61% (63/104), injection-site pain in 56% (58/104) and chills in 50% (52/104) [67]. The efficacy of this combination is currently being evaluated in a Phase 3 trial (NCT05933577).

Clinical investigations of mRNA-based TAA vaccines have shown promising results, with confirmatory studies ongoing. A Phase 1/2 study of the mRNA-4359 vaccine incorporating PD-L1 and IDO1 epitopes in combination with pembrolizumab in 29 patients with ICI-refractory metastatic melanoma demonstrated an ORR of 24% and a disease control rate (DCR) of 60%, with a higher response seen in PD-L1 TPS ≥1% tumours (ORR 67%). The median duration of response was not reached as the Phase 2 trial is ongoing [68].

Another off-the-shelf fixed liposomal mRNA vaccine (BNT111) incorporating four non-mutant shared TAAs (MAGE-A3, NY-ESO-1, tyrosinase, TPTE) has been evaluated in a Phase I/II study [69] in ICI-resistant, advanced melanoma patients expressing at least one of the antigens. The fixed mRNA vaccine was given alone or in combination with cemiplimab, an anti-PD-1 antibody. At the most recent update from the Phase II study, with a median follow-up of 15.6 months, the combination of BNT111 and cemiplimab demonstrated an mPFS of 3.1 months, mOS of 20.7 months, ORR of 18.1% and DCR of 55.3%, compared to mPFS, mOS, ORR and DCR of 2.8 months, 13.7 months, 17.4% and 58.7% in the BNT111 monotherapy arm, and 3.2 months, 22.3 months, 13.6% and 47.7% in the cemiplimab monotherapy arm. Of note, 48.9%, 43.5% and 50% of patients in the combination, BNT111 monotherapy and cemiplimab monotherapy arms, respectively, were anti-CTLA-4 pre-treated. The study demonstrated clinical activities of BNT111 monotherapy and a statistically significant improvement on the pre-defined assumed historical control ORR of 10%. Low-grade pyrexia (76.1%) and chills (56.5%) were the main side effects associated with the vaccine, thought to be driven by cytokine inductions through TCRs by the single-stranded RNAs. In total, 46.7% of patients experienced Grade 3 TRAEs in the combination arm, with 18.5% and 14.1% attributable to the vaccine and cemiplimab, respectively [70].

### 4.2. SLP Vaccines

Historically, short peptide vaccines consisting of single tumour antigen epitopes have failed to demonstrate significant clinical efficacy, likely because they lack strong immunogenicity and cross-presentation by professional APC, which leads to insufficient cytotoxic T-cell and CD4+ T-helper cell activation to sustain T-cell effector function [12,17]. Furthermore, short peptide vaccines are HLA-restricted and do not accommodate the high HLA polymorphism in the general population [17]. For example, gp100:209-217(210M), which is an 8-amino acid peptide vaccine, was investigated in two Phase III melanoma studies. In one study [71], the gp100 peptide vaccine was evaluated in combination with ipilimumab, an anti-CTLA-4 antibody, versus ipilimumab monotherapy in 676 patients with Stage IV and unresectable Stage III melanoma. The combination therapy did not show any additional survival benefit compared to ipilimumab, with an mOS of 10.0 months in the combination group, 6.4 months in the gp100 monotherapy group, and 10.1 months in the ipilimumab monotherapy group (HR for death 0.66 comparing ipilimumab monotherapy versus gp100 monotherapy; *p* = 0.003). In the other study [72], the gp100 peptide vaccine was investigated in combination with IL-2, compared to IL-2 alone, in unresectable Stage III and IV melanoma. When given in combination with IL-2, the vaccine combination demonstrated a significantly improved ORR (16% versus 6%, *p* = 0.03) and survival benefit (mPFS 2.2 months versus 1.6 months, *p* = 0.008; mOS 17.8 months versus 11.1 months, *p* = 0.06) compared to IL-2 alone. However, in vitro analysis showed that there was no correlation between anti-peptide activity and objective clinical response in the vaccine arm, and post-treatment increase in T regulatory cells was likely due to the effect of IL-2 and not the vaccine. Enthusiasm was also tempered by the significantly high-grade toxicity rates associated with IL-2 therapy [13]. Additionally, both trials also required patients to be HLA*A0201 positive.

MAGE-A3 is another TAA commonly associated with melanoma and was investigated in two melanoma vaccine studies. A Phase 2 study of MAGE-A3 peptide protein administered with immunostimulants eAS02B (TLR-4 agonist) or AS15 (TLR-9 agonist) in MAGE-A3-positive Stage III or IV M1a melanoma patients demonstrated modest activity. AS15 was found to induce a higher immunological and clinical response compared to AS02B, with the MAGE-A3-AS15 arm achieving an ORR of 11.1%, an mPFS of 2.9 months, and an mOS of 33 months [73]. However, the Phase 3 DERMA trial of the MAGE-A3-AS15 vaccine as adjuvant therapy in resected, MAGE-A3-positive Stage III melanoma failed to improve RFS in the MAGE-A3 group compared to the placebo group (HR 1.01; 95% CI 0.88–1.17; *p* = 0.86). Whilst the vaccine is composed of a recombinant MAGE-A3 protein, it failed to induce T-cell responses (particularly CD8+ responses), suggesting potential failure in antigen selection, the low immunogenicity of the full-length antigen, or limitations of single-antigen vaccines [74,75]. Other studies showed similarly disappointing results [13,72].

In contrast, SLPs (11–30 amino acids) incorporating multiple epitopes presented on both MHC I and MHC II molecules can elicit a coordinated CD4+ and CD8+ T-cell response, leading to a robust and sustained anti-cancer immune response. Furthermore, SLP vaccines that include multiple epitopes against various targets are more likely to overcome immune escape caused by antigen loss [7,76,77]. Additionally, SLPs have been shown to be more immunogenic than the full-length protein from which they are derived [75].

In a recent Phase I/II study [78], an off-the-shelf SLP vaccine incorporating dual IDO and PD-L1 epitopes of 21 and 19 amino acids, respectively, was administered in combination with nivolumab, an anti-PD-1 antibody, in metastatic melanoma patients who were anti-PD-1 naïve (Cohort A) and in patients who had progressed on anti-PD-1 therapy (Cohort B). In Cohort A, an ORR of 80% and a complete response (CR) rate of 50% were observed. At a median follow-up of 45.3 months, mPFS was 25.5 months, and mOS was not reached. In Cohort B, mPFS was 2.4 months and mOS was 16.7 months. This reduced efficacy in the setting of anti-PD-1 resistance was attributed to the production of dysfunctional PD-1+CD38hi CD8+ cells [79].

In the confirmatory Phase III study of upfront IDO/PD-L1 vaccine (IO102-IO103) in combination with pembrolizumab versus pembrolizumab alone in advanced melanoma, the primary endpoint of mPFS narrowly missed statistical significance (19.4 months vs. 11.0 months; HR 0.77; 95% CI 0.58–1.00; *p* = 0.0558). Clinical benefits were observed in patients without prior neoadjuvant/adjuvant anti-PD-1 treatment (HR 0.75; 95% CI 0.56–0.98; *p* = 0.037), and critically in patients with PD-L1-negative tumours, where the mPFS was 16.6 months in the combination arm versus 3.0 months with pembrolizumab alone (HR 0.54; 95% CI, 0.35–0.85; *p* = 0.006), signaling a potential for vaccine combinations to overcome ICI resistance in PD-L1-negative disease. Translational data also demonstrated the vaccine’s ability to modulate the TME by increasing tumour and immune cell expression of PD-L1 and IDO, increasing T-cell clonal expansion and infiltration at tumour sites, thereby increasing response to anti-PD-1 therapy [80].

SLP vaccines can also be personalised to incorporate neoantigens; this is less advanced than the mRNA vaccine platform. In a Phase I study, long peptides encoding patient tumour-specific neoepitopes were incorporated into a vaccine, which was delivered as a monotherapy to eight patients with fully resected Stage IIIB/C or stage IVM1b melanoma. Persistent neoantigen-specific CD4+ and CD8+ responses were present at 4.5 years after vaccination. While none of the patients had radiologically measurable tumours at the time of vaccination, there was evidence of epitope spreading, which is indicative of tumour cell lysis, as it would have released additional neoantigens or TAAs, which can trigger additional tumour-specific immune responses. All patients were alive at the time of reporting; the vaccine-induced melanoma-free survival rate was 37.5%. Of those who relapsed following vaccine therapy, 60% were able to achieve CR with subsequent anti-PD-1 therapy or surgery [81,82]. KEYNOTE-D36 was a proof-of-concept Phase II trial that evaluated the personalised artificial intelligence (AI)-derived cancer vaccine EVX-01 with pembrolizumab in unresectable treatment-naïve Stage III/IV melanoma patients. Ranked tumour-specific neoantigens for individual patients were synthesised into SLP vaccines with a liposomal immune adjuvant (CAF09b) and administered following 12 weeks of pembrolizumab induction. At 2-year follow-up, the best ORR was 75%. Median duration of response was 21 months. 92% of responders continued to respond at the latest follow-up. All patients demonstrated potent neoantigen-specific CD4+ and CD8+ responses [83,84].

**Table 1:** Selected completed and ongoing clinical trials on peptide and mRNA vaccines in melanoma.

Trial ID	Phase	Target Antigen	Platform	Trial Design	Clinical Setting	Outcome
NCT00094653 [71]	Phase III	gp100	Peptide	3:1:1 randomisation—vaccine + ipilimumab: ipilimumab: vaccine	Metastatic	mOS 10.0 vs. 10.1 vs. 6.4 months ORR 5.7% vs. 10.9% vs. 1.5%
NCT00019682 [72]	Phase III	gp100	Peptide	1:1 randomisation—vaccine + IL-2:IL-2	Stage III–Stage IV M1c	mPFS 2.2 vs. 1.6 months mOS 17.8 vs. 11.1 months ORR 16% vs. 10% G3-5 toxicities: 86% vs. 80%
NCT00086866 [73]	Phase II	MAGE-A3	Peptide	1:1 randomisation—vaccine + AS15: vaccine + AS02B	Stage III–Stage IV M1a	ORR 11.1% vs. 2.8% mPFS 2.9 vs. 2.9 months mOS 33.0 vs. 19.9 months
NCT00796445 [74]	Phase III	MAGE-A3	Peptide	2:1 randomisation—vaccine + AS15: placebo	Resected Stage III	mDFS 11.0 vs. 11.2 months
NCT03047928 [78]	Phase I/II	IDO, PD-L1	Peptide	Cohort A: anti-PD-1 naïve Cohort B: prior anti-PD-1 exposure Both cohorts received vaccine + nivolumab	Metastatic	Cohort A: ORR 80%, mPFS 25.5 months, mOS NR Cohort B: ORR 0%, mPFS 2.4 months, mOS 16.7 months
NCT05155254 [80]	Phase III	IDO, PD-L1	Peptide	Vaccine + pembrolizumab vs. pembrolizumab	Unresectable Stage III–Stage IV M1d	mPFS: 19.4 months vs. 11.0 months; HR 0.77; 95% CI 0.56–0.98; *p* = 0.056 mPFS in PD-L1 negative: HR 0.54; 95% CI, 0.35–0.85; *p* = 0.006
NCT01970358 [81]	Phase I	Up to 20 neoantigens	Peptide	Single-arm study	Resected Stage IIIb–Stage IV M1b	Persistent neoantigen-specific T-cell response present 4.5 years after vaccination. 37.5% pts remained melanoma-free.
NCT05309421 [83]	Phase II	7–10 neoantigens	Peptide	Singel arm study	Unresectable Stage III/IV	2-year follow-up: 44% PR, 37.5% SD, 12.5% PD, 6% CR
NCT02410733 [69]	Phase I/II	MAGE-A3, NY-ESO-1, tyrosinase, TPTE	mRNA	7 dose escalation and 3 dose expansion cohorts, latter with vaccine alone vs. in combination with anti-PD1	ICI pre-treated Stage IIIb–Stage IV	Vaccine alone (*n* = 25): 3 pts had PR, 7 pts had SD, 1 pt had CMR Vaccine/anti-PD1 (*n* = 17): 6 pts had PR
NCT04526899 [70]	Phase II	MAGE-A3, NY-ESO-1, tyrosinase, TPTE	mRNA	Vaccine + cemiplimab vs. vaccine vs. cemiplimab	ICI pre-treated unresectable Stage III–IV	ORR: 18.1% vs. 17.4% vs. 13.6% mPFS: 3.1 vs. 2.8 vs. 3.2 months mOS: 20.7 vs. 13.7 vs. 22.3 months
NCT02035956 [65]	Phase I	Up to 10 neoantigens	mRNA	Single-arm study	Stage III and Stage IV	Strong poly-neo-epitopic immune response against vaccine antigens was detected in all patients.
NCT03897881 [67]	Phase IIb	Up to 34 neoantigens	mRNA	2:1 randomisation—vaccine + pembrolizumab:pembrolizumab	Resected Stage IIIb–Stage IV	HR for recurrence or death 0.561 [95% CI 0.309–1.017] 18-month RFS: 79% vs. 62%
NCT05933577	Phase III	Up to 34 neoantigens	mRNA	Vaccine + pembrolizumab vs. pembrolizumab	Resected Stage II–IV	Ongoing

CMR: complete metabolic remission; CR: complete response; ICI: immune checkpoint inhibitor; mDFS: median disease-free survival; mOS: median overall survival; mPFS: median progression-free survival; ORR: overall response rate; PD: progressive disease; PR: partial response; pts: patients; RFS: recurrence-free survival; SD: stable disease.

## 5. Neoadjuvant Cancer Vaccines in Melanoma—The Way Forward?

The presence of tumour antigens is a prerequisite for effective immune cell activation. While surgical resection of tumours offers clear therapeutic benefit, it simultaneously removes the majority of the tumour antigens, potentially diminishing immune activation, even in the presence of immunotherapy. Administering immunotherapy in the neoadjuvant setting, when the tumour bulk and corresponding draining lymph node basins are in situ, has been shown to induce a stronger and more diverse T-cell response, leading to significantly improved clinical outcomes [85,86].

Several landmark randomised trials, most notably SWOG1801 [6], a Phase II trial of perioperative pembrolizumab versus adjuvant pembrolizumab, and the Phase III NADINA trial of neoadjuvant ipilimumab and nivolumab versus adjuvant nivolumab, have firmly established neoadjuvant ICIs as a new standard of care in melanoma [87,88,89]. In SWOG1801, the 3-year event-free survival (EFS) was 68% in the neoadjuvant pembrolizumab arm, compared to 56% in the adjuvant pembrolizumab cohort (HR 0.67; 95% CI 0.42–0.94) [90]. Similarly, NADINA showed a 2-year EFS of 77.3% in the neoadjuvant ipilimumab and nivolumab arm and 55.7% in the adjuvant nivolumab arm (HR 0.40; 95% CI 0.28–0.57; *p* < 0.001). For patients who achieved a pathological complete response following neoadjuvant therapy, the 2-year EFS was 95.1% [91].

One of the important outcomes of the NADINA study, building on the findings from the PRADO study [88], was the validation of a response-directed adjuvant therapy strategy. In both studies, patients who achieved a major pathological response to neoadjuvant ICIs, defined as <10% viable tumour at surgical resection, were able to forgo additional adjuvant ICIs following surgery without compromising EFS. This approach supports the personalisation of adjuvant treatment based on tumour response assessment, allowing some patients to avoid unnecessary exposure to adjuvant ICI while maintaining excellent efficacy.

Given the mechanism of cancer vaccines, one could hypothesise that introducing cancer vaccines into the neoadjuvant setting could further enhance immune response and clinical benefit [92]. For instance, an adenovirus-based vaccine encoding tyrosinase-related protein 2, which is widely expressed in melanoma, was tested in preclinical models and found to confer protection against tumour recurrence when given before surgery, while no benefit was observed when given after surgery [93]. However, one of the challenges with neoadjuvant vaccine delivery is the lengthy manufacturing process, which may not be practical when there is urgency to treat the malignancy in the neoadjuvant setting. In KEYNOTE-942, the median time from surgery to the first dose of pembrolizumab was 10 weeks, and the majority (81%) of the patients received the first dose of vaccine with cycle 3 pembrolizumab, approximately 6 weeks after the initial dose of pembrolizumab [67].

The KEYNOTE-942 study recruitment spanned the years of the COVID-19 pandemic, and prioritisation for COVID-19 vaccine manufacturing during the pandemic may have partly impacted the manufacturing capacity of the cancer vaccine. However, dedicated production lines and marked improvements in mRNA and protein production technologies are shortening the vaccine production timelines, potentially overcoming this barrier. One question that remains is the optimal number of vaccine doses required in the neoadjuvant setting for an effective response. A Phase II/III study (NCT06295809) is also currently underway to investigate the efficacy and safety of neoadjuvant-adjuvant pembrolizumab and the personalised mRNA V940 vaccine in cutaneous squamous cell carcinoma. In the pembrolizumab/V940 arm, patients receive two doses of pembrolizumab delivered every 6 weeks and two doses of the V940 vaccine every 3 weeks before surgery [94]. A Phase II study (NCT05280314) investigating an off-the-shelf peptide vaccine (IO102-IO103) in combination with pembrolizumab as neoadjuvant-adjuvant therapy for resectable melanoma is also currently underway. Beyond assessing efficacy, these studies will provide important proof of logistic feasibility for delivering cancer vaccines in the neoadjuvant context.

Overall, mRNA- or SLP-based cancer vaccines represent promising new frontiers in melanoma therapy, particularly in combination with ICIs. Advances in manufacturing technologies and ongoing clinical research are expected to address current logistical challenges and support their integration into the mainstream melanoma treatment paradigm.

## 6. Conclusions

Genomics-led personalised cancer vaccines represent a rapidly evolving frontier in melanoma treatment. mRNA and SLP vaccine technologies offer clinically viable approaches to modulate the tumour immune microenvironment, improve the effectiveness of ICIs, and generate robust antitumour responses to both tumour-associated antigens and neoantigens, with minimal additional systemic toxicity. The success of neoadjuvant ICI therapies in melanoma underscores the potential for vaccines to further improve outcomes in this setting. The main barrier to neoadjuvant implementation is the timely manufacture of a vaccine to be suitable for the neoadjuvant window, an obstacle likely to be overcome through continued optimisation of mRNA and peptide vaccine platforms.

## Data Availability

No new data were created or analysed in this study.

## Abbreviations

APC	antigen-presenting cell
CI	confidence interval
CR	complete response
CTLA-4	cytotoxic T-lymphocyte-associated protein 4
DCR	disease control rate
EFS	event-free survival
gp100	glycoprotein-100
HLA	human leukocyte antigen
HR	hazard ratio
ICI	immune checkpoint inhibitor
INDEL	single-nucleotide base insertions or deletions
LNP	lipid nanoparticle
MAGE	melanoma antigen gene
MFS	metastasis-free survival
MHC	major histocompatibility complex
mRNA	messenger RNA
NGS	next-generation sequencing
ORF	open reading frame
ORR	overall response rate
OS	overall survival
PD	progressive disease
PD-1/PD-L1	programmed death-1/programmed death-ligand 1
PFS	progression-free survival
PRAME	preferentially expressed antigen in melanoma
RFS	relapse-free survival
SD	stable disease
SLP	synthetic long peptide
SNV	single-nucleotide variant
TAA	tumour-associated antigen
TCR	T-cell receptor
TIL	tumour-infiltrating lymphocytes
TMB	tumour mutation burden
TRAE	treatment-related adverse event
Treg	regulatory T cell
TSA	tumour-specific antigen
UV	ultraviolet
